# Prevalence of extensive drug resistance in bacterial isolates harboring *bla*NDM-1 in Quetta Pakistan

**DOI:** 10.12669/pjms.35.4.372

**Published:** 2019

**Authors:** Mohammad Din, Khan M. Babar, Shabir Ahmed, Abdul Aleem, Dawood Shah, Dawood Ghilzai, Nazeer Ahmed

**Affiliations:** 1 Mohammad Din PhD Scholar (Microbiology), Department of Pathology/General, Neuro and Cardiac Surgery/ Gastroenterology, Bolan Medical College/Complex Hospital, Quetta, Pakistan; 2 Dr. Khan Mohammad Babar, FCPS, Department of Pathology/General, Neuro and Cardiac Surgery/ Gastroenterology, Bolan Medical College/Complex Hospital, Quetta, Pakistan; 3 Dr. Shabir Ahmed Lehri, FCPS, Department of Pathology/General, Neuro and Cardiac Surgery/ Gastroenterology, Bolan Medical College/Complex Hospital, Quetta, Pakistan; 4 Abdul Aleem, MS, Department of Pathology/General, Neuro and Cardiac Surgery/ Gastroenterology, Bolan Medical College/Complex Hospital, Quetta, Pakistan; 5 Dr. Dawood Shah, FCPS, Department of Pathology/General, Neuro and Cardiac Surgery/ Gastroenterology, Bolan Medical College/Complex Hospital, Quetta, Pakistan; 6 Dr. Dawood Ghilzai, FCPS, Department of Pathology/General, Neuro and Cardiac Surgery/ Gastroenterology, Bolan Medical College/Complex Hospital, Quetta, Pakistan; 7 Dr. Nazeer Ahmed, PhD, Balochistan University of Information Technology, Engineering and Management, Sciences, Quetta, Pakistan

**Keywords:** Carbapenemase, Modified Hodge Test, New Delhi-Metallo-Beta-Lactamase, PDR, XDR. CRE

## Abstract

**Objective:**

Extensive drug resistant Gram-negative bacilli, harboring New Delhi metallo-β-lactamase-1 (*bla*_NDM-1_) having the ability to hydrolyze β-lactams, have become a vital global clinical threat. The present study was, therefore, designed to investigate the prevalence and epidemiology of NDM-1 producers in Quetta, Pakistan.

**Methods:**

This study was carried out in Microbiology Laboratory, Bolan Medical Complex Hospital Quetta, Biotechnology laboratory, BUITEMS Quetta and Hi-tech laboratory, CASVAB, University of Balochistan, Quetta, from March to June 2018, during the hot season. Biochemical and molecular approaches were applied for the identification of bacterial isolates. Minimum Inhibitory Concentrations (MICs) were determined using E-test method. Carbapenemase activity was ascertained by Modified Hodge Test (MHT) and the presence of *bla_NDM-1_* gene was recognized by Polymerase Chain Reaction (PCR).

**Results:**

We isolated five *bla*_NDM-1_ harboring isolates of three different species namely *Morganella morganii* (n=2) *Enterobacter cloacae* (n=2) and *Citrobacter freundii* (n=1), from 300 pus samples. These isolates were found extensive drug resistant (XDR). Strikingly, two isolates of *M*. *morganii* were displaying resistance against 23 antibiotics of sulphonamides, aminoglycosides, polypeptide, monobactams, tetracyclines, quinolones, macrolides, cephalosporins, phosphonic acid and β-lactams groups, suggesting Pan Drug Resistance (PDR).

**Conclusion:**

This is the first report on emergence of PDR strain of *M. morganii* producing NDM-1 in the province of Balochistan, Pakistan. The presence of *bla*_NDM-1_ in different bacterial species and their extensive rather pan drug resistance pattern poses a momentous clinical threat.

## INTRODUCTION

Carbapenem group of antibiotics is one of the most effective drugs that has been used as a last remedy for the treatment of infections caused by Multi Drug Resistant (MDR) Gram negative bacilli.^1^ To neutralize the drugs, such NDM-1 producing bacteria encode various carbapenemases which are β-lactamases, having the ability to hydrolyze majority of β-lactams including carbapenems.^2^ Irrational use of antimicrobials give birth to resistance by increasing selective pressure in the bacterial population. According to the latest reports on antimicrobial resistance β-lactamases are suggested to contribute to resistance.^3^ *M. morganii* and *E. cloacae* which are members of *Enterobacteriaceae* family play a significant role in causing nosocomial infections like, wounds, skin, soft tissue, urinary tract, respiratory tract, and biliary tract, mostly in immunocompromised patients. NDM-1 was reported from different hospitals in the UK in *Morganella morganii, Citrobacter freundii, Enterobacter cloacae, Providencia, Klebsiella* *pneumonia* and *Escherichia coli*.^4^,^5^ It was initially discovered in 2008 in a Swedish patient of Indian origin, but now has claimed its presence globally including India and Pakistan.^5^,^6^

United Kingdom, Canada, France, China, Japan, Oman, Iraq, Bangladesh and United States are one of the few other countries from where *bla*_NDM-1_ has been reported.^6^ The emergence of carbapenem resistance, mostly associated with *Enterobacteriaceae*, raised with the ability of rapid clonal distribution due to its presence on mobile elements such as plasmids.^5^,^7^ There are random reports of the isolation of *bla*_NDM-1_from different parts of Pakistan.^5^,^8^,^9^ However, such studies have never been reported from the province of Balochistan. The purpose of this study was therefore, to unleash the prevalence and epidemiology of NDM-1 producers from clinical samples in Quetta, Pakistan.

## METHODS

Three hundred patients of both genders, adults and children from surgical wards, burn wards, and burn intensive care units (ICU’s) of three tertiary care hospitals (Bolan Medical Complex Hospital, Sandeman Provincial Hospital and Fatima Jinnah Chest Hospital) in Quetta were selected in this study. Detailed history was taken from each patient regarding, wound, time and duration of pus discharge, previous antibiotic therapy, pre-existing clinical complications and the areas/climate from where they belonged to. Those patients were preferred for the study whose pus was still oozing from their wounds after several antibiotic therapies. Antibiotic therapy of the patients was discontinued for 72 hours before sample collection.This study was carried out in Microbiology Laboratory, Bolan Medical Complex Hospital Quetta, Biotechnology laboratory, BUITEMS Quetta and Hi-tech laboratory, CASVAB, University of Balochistan, Quetta, from March to June 2018, during the hot season.

### Ethical considerations

Ethical approval for the present study was taken from the Institutional Bioethical Committee Bolan Medical Complex Hospital, PMRC R.No G 263 Quetta Balochistan.

### Sample collection

Pus samples in sterile 5cc B.D syringes (USA) and with sterile cotton swabs (prepared in lab) from surgical/burn wounds of the patients were taken aseptically. The surface area of the wound was cleaned with sterilized cotton. Efforts were made to collect fresh pus from inside the wound after applying slight pressure. Samples were labeled and transported to the Microbiology laboratory immediately. All the samples were inoculated within one hour of collection.

### Phenotypic and genotypic characterization of bacterial isolates

Conventional microbiological procedures were adopted for bacterial isolation from pus samples. Each sample was streaked simultaneously on MacConkey and Blood agar plates (Oxoid, United Kingdom) followed by incubation aerobically at 37°C for 24 hours.10 Plates were observed for bacterial colonies and the isolated colonies were further triple cloned. Isolates were identified by analytical profile index, API 20E system (bioMerieux, France) according to the manufacturer’s instructions (http://www.biomerieux-usa.com/clinical/api). Thermo Scientific Genomic Purification Kit, Lithuania, was used for DNA extraction, according to the manufacturer’s instructions. Internal fragment of 1500 bp of 16S rDNA gene was amplified using universal primers, 27F-5’- AGA GTT TGA TCC TGG CTC AG -3’ and RD1-5’- AAG GAG GTG ATC CAG CC -3’ with Initial denaturation 95°C for 2 min, followed by 35 cycles of 30 sec. at 95°C, 30 sec. at 55°C and 2 min at 72°C. Final extension was set at 72°C for 10 min.11

### Susceptibility testing

Antibiogram was performed on Mueller-Hinton agar plates using disc diffusion Kirby Bauer technique and 0.5 McFarland turbidity standard methods. Interpretation of the results was based on the Clinical and laboratory Standards Institute (CLSI) guidelines.^12^ The MICs were performed by the E-test method (Oxoid UK and Liofilchem Italy). Interpretation of the results was based on CLSI^13^ and Food and Drug Administration (FDA).^14^ breakpoints and recommendations.

### Identification of carbapenemase producers

Phenotypic detection of carbapenemase enzyme activity of bacterial isolates was achieved by Modified Hodge Test (MHT), as described by (CLSI).^12^ American Type Culture Collection (ATCC) strains of *Klebsiella pneumoniae* BBA 1706 (negative control), *Klebsiella pneumoniae* BBA 1705 (positive control) and *Escherichia coli* 25922, were used in the assay.

### Amplification of bla_NDM-1_ producers:

Plasmid DNA was extracted for selected phenotypically carbapenems resistant isolates using GeneJET Plasmid Miniprep Kit by Thermo Fisher Scientific Lithuania, according to the manufacturer’s instructions. The *bla*_NDM-1_ gene was amplified with the following set of primers; *bla*_NDM-1_ F-5’- GGG CAG TCG CTT CCA ACG GT-3’ and *bla*_NDM-1_ R-5’- GTA GTG CTC AGT GTC GGC AT -3’. Conditions for PCR were set to; initial denaturation at 95°C for 5 min, followed by 30 Cycles of 95°C for 40 sec, 58°C for 30 sec, 72°C for 30 sec with final extension 72°C for 5 min.^15^

### Sequencing and analysis:

Sequencing of the PCR product of 16S rDNA and *bla*_NDM-1_ genes of the representative samples was carried out commercially through Macrogen, South Korea. Deduced sequences were aligned using Basic Local Alignment Search Tool (BLAST). NCBI at (https://blast.ncbi.nlm.nih.gov/Blast.cgi).

## RESULTS

### Overall abundance of bacterial isolates from pus samples:

**Fig. 1:**
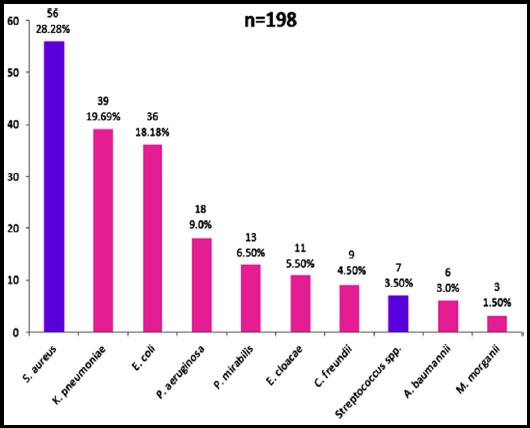
Number & percentage of different bacterial isolates in 198 pus samples.

Of the 300 pus samples, 102 (34%) showed no bacterial growth whereas, 198 (66%) exhibited bacterial growth on MacConkey and blood agar plates after aerobic incubation at 37°C for 24 hrs. Based on cultural, biochemical and morphological characterization, and using API system, 10 different bacterial types were tentatively identified, which included *Staphylococcus aureus* (n=56) and *Klebsiella pneumoniae* (n=39) as predominant, while *Acinetobacter baumannii* (n=6) and *Morganella morganii* (n=2) were the least dominant (Fig.1).

### Susceptibility testing

**Fig. 2:**
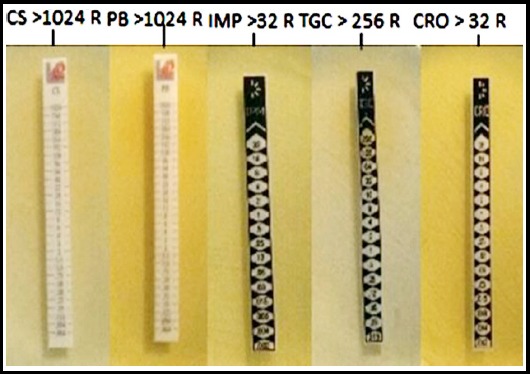
MICs of PDR M. morganii.

All Gram-negative isolates of different spp. subjected to antibiogram were sensitive to a range of antibiotics except, *Morganella morganii*, (n=2), *Enterobacter cloacae* (n=2) and *Citrobacter freundii* (n=1). These five isolates were tested against twenty-three available antibiotics from, aminoglycosides, tetracyclines, quinolones, β-lactams, monobactams, sulphonamides, macrolides, cephalosporins, phosphonic acid and polypeptide classes of antimicrobials, showed increased resistance. Strikingly two isolates of *M. morganii* (Mm-141, and Mm-234) exhibited an extreme level of resistance against β-lactam and non β-lactam classes of antimicrobials, whereas out of 23 antibiotics *Enterobacter cloacae* (Ec-93 and Ec-188) were sensitive to tigecycline and *Citrobacter freundii* (Cf-276) to polymyxin-B, tigecycline and colistin only. The resistance pattern of these isolates indicated that these are XDR and/or PDR (Table-I and Fig.2).

**Table I:** Susceptibilities of blaNDM-1-producing isolates.

MICs (µg/dL)						

Isolate	IPM	PB	TGC	CS	CRO	Other resistance markers (Disc diffusion Method)
Ec-93	>32 R	12 R	0.25 S	16 R	>32 R	ETP, MEM, CIP, SXT, AMC,TZP,AK,CN, AMP,TE,CE,FOS,CLR,EN,CTX,OFX,CXM,CEC
Mm-141	>32 R	>1024 R	>256 R	>1024 R	>32 R	ETP, MEM, CIP, SXT, AMC,TZP,AK,CN,AMP, TE,CE, FOS,CLR,EN,CTX,OFX,CXM,CEC
Ec-188	16 R	16 R	0.06 S	8 R	>32 R	ETP, MEM, CIP, SXT, AMC,TZP,AK,CN,AMP, TE,CE,FOS,CLR,EN,CTX,OFX,CXM,CEC
Mm-234	>32 R	>1024 R	>256 R	>1024 R	>32 R	ETP, MEM, CIP, SXT, AMC,TZP,AK,CN,AMP, TE,CE, FOS,CLR,EN,CTX,OFX,CXM,CEC
Cf-276	>32 R	0.38 S	0.12 S	0.75 S	>32 R	ETP, MEM, CIP, SXT, AMC,TZP,AK,CN,AMP, TE,CE, FOS,CLR,EN,CTX,OFX,CXM,CEC

**Ec:** Enterobacter cloacae; **Mm:** Morganella morganii; **Cf:** Citrobacter freundii, **IMP:** imipenem;

**PB:** polymyxin B, **TGC:** tigecycline, **CS:** colistin; **CRO:** ceftriaxone, **ETP:** ertapenem, **MEM:** meropenem, **CIP:** ciprofloxacin, **SXT:** trimethoprim/sulfamethoxazole, **AMC:** amoxicillin/clavulanic acid, **TZP:** piperacillin/tazobactam, **AK:** amikacin, **CN:** gentamycin, **AMP:** ampicillin, **TE:** tetracycline, **CE:** cephradine, **FOS:** fosfomycin, **CLR:** clarithromycin, **EN:** enoxacin, **CTX:** cefotaxime, **OFX:** ofloxacin, **CXM:** cefixime, **CEC:** cefaclor.

### Phenotypic identification of carbapenemase producers

All five isolates, *Morganella morganii*, (n=2), *Enterobacter cloacae* (n=2) and *Citrobacter freundii* (n=1), were subjected to MHT for phenotypic detection of carbapenemase enzyme activity as described by CLSI.^12^ Out of 5 *bla***_NDM-1_** producers, four gave positive clover-leaf like indentation whereas, isolate Ec-93 was negative. The sensitivity of the test was 80 %.

### 16S rDNA gene-based identification of bla_NDM-1_ producers

All five suspected *bla***_NDM-1_** producers were successfully amplified for 16S rDNA gene. The amplified products (1500 bp) of the representative isolates were sequenced. The obtained sequences were BLAST to ascertain the molecular based identification and confirmation of the isolates. The retrieved sequences, as a result of BLAST, showed 100 % similarity to the previously reported genes. https://blast.ncbi.nlm.nih.gov/Blast.cgi

### Molecular detection of bla_NDM-1_ gene

Phenotypically five XDR and/or PDR isolates were amplified by PCR for *bla***_NDM-1_** gene. An amplicon size of 475 bp confirmed the existence of *bla*_NDM-1_ gene in the tested isolates^15^. Importantly, isolate that gave negative MHT result was also found harboring *bla***_NDM-1_** gene. Representative PCR amplified products were sequenced. The retrieved sequences, as a result of BLAST, showed 99% similarity to the previously reported *bla***_NDM-1_** genes. https://blast.ncbi.nlm.nih.gov/Blast.cgi

## DISCUSSION

New Delhi Metallo- β-lactamase-1 (*bla***_NDM-1_**), which was initially reported in a Swedish patient of Indian origin in 2008,^5^ has become an alarming clinical threat and economical burden particularly in developing countries like Pakistan, India and Balkan.^16^ Since then it has spread globally with a continuous increase in many countries of Asia, Europe, America, Australia and Africa. 17

In the present study we isolated five extensive and/or pan drug resistant NDM-1 producers belonging to carbapenem resistant *Enterobacteriaceae* (CRE) of three different spp. from three hundred pus samples. *bla*_NDM-1_gene was detected in *Morganella morganii*, (n=2), *Enterobacter cloacae* (n=2) and *Citrobacter freundii* (n=1). Of all *Enterobacteriaceae* in our study, NDM-1 was present in 4.27 % as opposed to 2.7% in Kuwait, 1.2% reported in Pakistan, India and the United Kingdom and 1.1% reported in Vietnam.^5^,^18^ This increase in the prevalence of NDM-1 is alarming in the region.

Multi drug resistance was observed in NDM-1 and NDM-5 variants that exhibited resistance to ciprofloxacin, amikacin and aztreonam, and pan drug resistance in other variants like NDM-4, NDM-7 and NDM-9 that showed resistance to nearly all antimicrobials used in clinical practice excluding tigecycline and colistin.^19^ In the present work all our isolates harboring NDM-1 variant were showing resistance to ciprofloxacin, amikacin and aztreonam, and *Morganella morganii* to tigecycline and colistin as well.

In an epidemiological report the occurrence of carbapenem resistance in the Asian countries from 2002-2012 was 2.4% to meropenem and 1.9% to imipenem,^20^ and in a study from Pakistan in 2018 was 56%.^21^ The resistance pattern of *Morganella morganii*, (67%), *Enterobacter cloacae* (18%) and *Citrobacter freundii* (11%) to the carbapenems was quite variable in our case.

In Southwest China resistance pattern of *Enterobacter cloacae* (CRE) was substantial against meropenem, ciprofloxacin, gentamycin, imipenem, tobramycin, levofloxacin and cefepime. Remarkably 65.9% were kept in the category of Multi Drug Resistant (MDR), use of their resistance against three or more antibiotic groups.^22^ Different observations were recorded in the present effort where isolate-Ec-93 and 188 (*E. cloacae*) were sensitive to tigecycline but resistant to polymyxin-B and colistin, showing probable genetic diversity of the isolates.

*Morganella morganii* indeed show resistance to polymyxin B and E, when isolated from blood and urine specimens was efficiently treated by combination therapy of fosfomycin in double dose and meropenem. 23 Isolate Mm-4232 showed resistance to β-lactams together with carbapenems, in combination with trimethoprim–sulfamethoxazole and aminoglycosides, was kept in the category of extensive drug resistance. Only fosfomycin was left as last choice of treatment. 23 Regarding sensitivity pattern, in 170 non-β-lactam antimicrobials, polymyxin-B was (83%), colistin (83%) and tigecycline (98%).^24^ However, isolates of the same microbe (Mm-141 and Mm-234) in our investigations displayed outstanding resistance against twenty-three available antibiotics belonging to cephalosporins, monobactams, quinolones, tetracyclines, β-lactams, aminoglycosides, macrolides, phosphonic acid, penicillins, polypeptides and sulphonamides classes, including the ones mentioned in the above studies.

*Citrobacter freundii* WCHCF65 isolated from hospital sewage showed resistance to meropenem, ciprofloxacin, imipenem and ceftazidime and was sensitive to tigecycline, colistin and amikacin,^25^ whereas isolate Cf-276 of the same sp. in the present study was sensitive to colistin and tigecycline but resistant to amikacin showing extensive resistance pattern and genetic diversity. MICs of imipenem, tigecycline, polymyxin-B, colistin and ceftriaxone exhibited extensive and pan resistance pattern. Viewing sensitivity pattern, these carbapenemase producers may be kept in the category of XDR and/or PDR.

The presence of NDM-1 in diverse microbial species necessitates precise and early detection particularly in view of the limited treatment options available. Addressing the issue, our investigations clearly suggest PCR assays more sensitive (100% sensitivity). than Modified Hodge test (80% sensitivity) in case of NDM-1 carbapenemase producers.

To the best of our knowledge, this is the maiden report on NDM producers in Balochistan, and pan drug resistant *Morganella morganii* in Pakistan. The present study not only revealed the presence of three different bacterial species harboring *bla***_NDM-1_** in the population of Balochistan, the largest province of Pakistan, but also profiled their extensive and pan drug resistance pattern against a range of antibiotics including carbapenems.

## CONCLUSION

Our study which intended to interpret the first detection, prevalence and epidemiology of NDM-1producers in clinical samples from Quetta, Pakistan, indicated five isolates harboring *bla*_NDM-1_, showing resistance to a range of antibiotics including carbapenems. Importantly, two isolates of *Morganella morganii* displayed resistance against all 23 antibiotics used in the study. The spread of *bla*_NDM-1_, extensive and pan drug resistance, lack of new antimicrobials particularly in carbapenem resistant *Enterobacteriaceae* (CRE) is an alarming clinical threat.

### Author`s Contribution

**MD, NA, KMB and DS** designed study outset and experiments, wrote the manuscript and contributed in biological, molecular experiments and sequencing data analysis.

**SA, AA, DG and MD** contributed in sample collection, data acquisition, interpretation and manuscript writing.

All authors collectively contributed to the critical review, significant rational amendment in the final manuscript and approval of the article to be published.
